# Thermal Analysis of Whole Bacterial Cells Exposed to Potassium Permanganate Using Differential Scanning Calorimetry: a Biphasic Dose-Dependent Response to Stress

**DOI:** 10.1100/tsw.2009.3

**Published:** 2009-02-15

**Authors:** Marina K. Abuladze, Victor M. Sokhadze, Emma N. Namchevadze, E. Kiziria, Leila V. Tabatadze, Lia V. Lejava, Sh. Gogichaishvili, Nugzar B. Bakradze

**Affiliations:** Department of Physics of Biological Systems, Andronikashvili Institute of Physics, 6 Tamarashvili str., Tbilisi, Georgia, 0177, Georgia

**Keywords:** differential scanning calorimetry, potassium permanganate, bacterial cell culture, Arthrobacter oxydans, stress response

## Abstract

Differential scanning calorimetry (DSC) was applied to estimate the impact of the toxic oxidant potassium permanganate (PM) on the intracellular structural and functional alterations at whole cell level using soil bacteria *Arthrobacter oxydans* as a model culture. Differential scanning calorimetry (DSC) was applied in order to estimate the impact of the toxic oxidant potassium permanganate (PM) on the intracellular structural and functional alterations at the whole cell level using the soil bacteria Arthrobacter oxydans as a model culture. We compared the total melting heat and the temperature of DNA-protein complex (DNP) melting at the PM application prior to the calorimetry measurement and after 24-h exposure at the concentration range 0.02–1.4 mM. The initial oxidative effect caused changes in the pattern of the whole cell melting spectra (mainly at the temperature range 56–78°C), the decrease of T_max_ °C DNP melting, and did not influence significantly the total heat of bacterial melting at different concentrations of PM. The prolonged effect of permanganate up to 24 h was characterized by a biphasic dose-dependent response to stress estimated by the DSC technique and the colony-forming assay. The low doses of PM (0.02 and 0.2 m*M*) stimulated cell proliferation, and increased the total whole cell melting heat and the temperature of DNP melting. The toxic effect of PM up to 0.04 m*M* reduced cell viability, changed the character of multipeaked thermograms, and lowered the total melting heat and the temperature of DNP melting in a concentration-dependent manner. This study presents the DSC method for evaluating and monitoring the effects of exposure to potential human and environmental toxicants.

### INTRODUCTION

Potassium permanganate (PM) is a broad-range oxidant that can convert many inorganic materials (such as iron, chromium, and sulfur ions/compounds) and organic compounds from lower oxidation states to higher oxidation states. The permanganate ion $MnO_4^{-2}$, derived from PM, is a strong oxidant and PM is used as a general biocide at recommended concentrations in various aquaculture settings[1,2,3]. Permanganate is applied for oxidizing low concentrations of organic and inorganic contaminants in water-treatment processes, and is utilized for soil remediation[4]. The primary mode of pathogen inactivation by PM is direct oxidation of cell material or specific enzyme destruction. Abiotic oxidation of the soil contaminants may have immediate or long-term adverse effects on microbial populations, and may induce biodegradability and/or toxicity to microorganisms. Effects on microbial viability revealed a community shift from a consortium composed predominantly of aerobic heterotrophs, anaerobic heterotrophs, nitrate-reducing bacteria, sulfate-reducing bacteria, and methanogens to primarily aerobic heterotrophs[4,5]. Permanganate is known to react preferentially with single-stranded DNA, and to detect promoter DNA melted in an open complex both *in vitro* and *in vivo*[6]. Phospholipids within the cell membrane, containing unsaturated fatty acids, may be susceptible to $MnO_4^-$ oxidation at carbon-carbon double bonds that results in formation of α-hydroxyketones, diols, and/or epoxides. Such reactions may induce loss in membrane function and cell death. Polysaccharides composing the peptidoglycan cell wall may be oxidized and removed, resulting in loss of cell wall stability and structure[4].

The permanganate multiple impact on bacterial cells may be estimated by differential scanning calorimetry (DSC), a bioanalytical technique that provides direct information of the conformation changes of biomolecules and other intracellular processes by measuring the melting process of different structural components of a cell. It has been applied to characterize various biological systems on the basis of the thermostability of cellular constituents, and to study biological responses to endogenous and exogenous stimuli[7,8,9]. The DSC method can be used to assess functional cellular alterations and perturbations in cellular systems at toxic compounds action on the whole cell level[10,11].

This paper describes the application of the DSC technique for the evaluation of dose- and time-dependent toxic effects of PM *in vivo* using widely spread soil bacteria of *Arthrobacter* sp., namely *A. oxydans,* as a model culture.

### MATERIALS AND METHODS

This study was conducted using soil *A. oxydans,* kindly provided by Dr. X.-Y. Holman (Lawrence Berkeley National Laboratory, Berkeley, CA), as a bacterial cell model system. *A. oxydans* was cultured in the standard medium recommended for *A. oxydans* sp.[12] at 21°C under permanent shaking. Cells were grown in liquid media as a batch culture.

#### Cell Suspension Optical Density ($OD_{562}$) Determination

OD determinations were performed in 96-well plates using a scanning multiwell spectrophotometer at 560 nm; 200 µl of cell suspension were transferred to each well. The plates were immediately measured after three rounds of shaking (20 sec). The linear relationship between OD measurements and cell suspension concentrations was determined.

#### Morphological Analysis

A complex morphological cycle of *A. oxydans* at their transition from the logarithmic to stationary phase[13] was registered by a fluorescence microscope (LUMAM I-2, Russia) using intact acridine orange (AO) staining. AO staining was performed on unfixed cells directly on microscope slides (50 µg/ml) at room temperature for 5 min and then rapidly dried in the stream of warm air. Stained samples were visualized under immersion objective.

### The Colony Forming Viability Assay

For *A. oxydans* cell viability determination after treatment with different concentrations of PM (0.02, 0.2, 0.4, 6.0, and 1.2 m*M*), 100 µl of cell suspension after several serial tenfold dilutions (from $10^{-3}$ to $10^{-8}$) were exposed on agar plates. The number of colonies was counted on the third, fourth, and fifth days after cell inoculation on agar plates. The data are presented as colony forming units per milliliter (CFU/ml). Numbers of CFU/ml were calculated by multiplying the number of colonies, the dilution factor, and the volume of the total sample in proportion to the 100-µl sample taken for setting up the colony culture.

### The DSC Measurement

Calorimetric study was carried out by means of a modified device originally elaborated and designed in the Andronikashvili Institute of Physics of the Georgian Academy of Sciences[14]. The measurements were conducted at a sampling frequency of 1 point per 2 sec, at a heating rate of 35 K/h that gave a temperature resolution of 0.02 K. The accuracy of absolute temperature measurement was not less than 0.01 K over the temperature range 25–110°C. The calorimetric ratio in accordance with thermal capacity change (i.e., significant deviation from the baseline) is $10^{-5}$ J/K. With a time constant of 1 min, this enables one to resolve peak distance no less than 0.6 K. All DSC measurement conditions (buffer system, scanning rate) were selected so that only endotherms were observed.

For the calorimetry measurement, the cell suspension was centrifuged at 10,000 rpm for 10 min and the pellet was washed several times in the low ionic buffer solution at 7–7.5 pH (50 m*M* Tris, 0.1 *M* NaCl, 0.1 m*M* PMSF, 0.1 m*M* benzamidine) with subsequent centrifugation. The selected pH range did not influence the pattern of the thermal spectra. Afterwards, 0.2 ml of the pellet was placed into the stainless steel measurement cell. The consumption of the matter in the experiment was 5–15 mg of dry weight. The thermal spectra were thermograms measured in the temperature range 25–110°C and normalized by the dry weight of the sample after drying at 105 ± 2°C.

### Data Processing

The initial data processing (e.g., baseline approximation, calibration, and curve normalization) was conducted using our in-house software. The final analysis (including identification of spectra maximums, integration of curves, and chart plotting) was conducted using the program package Origin 6.0 (Microcal™ Software, Inc.).

## RESULTS AND DISCUSSION

The DSC analysis of the toxic effect of the high-oxidant PM at concentration range from 0.02 to 1.2 m*M* was applied to the stationary growth phase culture of *A. oxydans*. The two models of the chemical impact on bacterial cells were analyzed: (1) the permanganate was applied directly to the cell suspension at 48 h of growth prior to the calorimetry measurement; (2) the permanganate was introduced into the culture media at 24 h of growth (early stationary growth phase) with subsequent 24-h growth in the presence of the chemical and also analyzed 48 h after cell inoculation. The first of the studied models permits us to estimate the initial oxidative impact of PM on bacterial cell structural components in a concentration-dependent manner, whereas the second one reflects the stress response of bacterial culture.

Multiple measurements demonstrated that the thermal spectra profile depended on cell culture conditions and growth stage, and could characterize the cell functional state. *A. oxydans* is characterized by a complex morphofunctional cycle, altering cell shape from rods in the logarithmic phase to cocci in the stationary phase[13]. This phenotype transformation is accompanied by quantitative and qualitative changes during cell transition from the logarithmic to stationary phase. The DSC could lay out in sequence the complex series of denaturation events that take place when cells are heated. The thermograms of *Escherichia coli* during heating, at a rate of 10°C/min, yielded a complex series of overlapping endothermic peaks in which the major events have been identified by cell fractionation. The characteristic peaks were assigned to cell wall lipids, ribosomes, and DNA[15]. The phenotype transformation of *A. oxydans* during cell transition from the logarithmic to stationary phase was analyzed and differentiated by the DSC method[10]. The thermal spectrum of the endothermic melting of complex biological structures in intact cells over the temperature range 40–105°C significantly altered in the 40–90°C interval, reflecting the orderly changes in protein composition, RNA and ribosomes level, membrane and cell wall structural elements at different points of the growth cycle. The spectral changes in the 90–110°C region corresponded to DNA-protein (DNP) complex. The reproducible decrease of the melting temperature of the *A. oxydans* DNP complex of 0.3 K at cell transition from the logarithmic to stationary growth stage was detected, reflecting the structural changes of the complex[10].

We limited the study of the permanganate toxic effect to the definite growth stage of the bacterial cell culture. The cell transition to the stationary phase was controlled by spectrophotometric measurements ($OD_{560}$) and fluorescent microscope analysis (data not shown). Fig. 1A shows the typical multipeaked thermal spectra of *A. oxydans* at the stationary growth phase (48 h of growth). Fig. 1B demonstrates the dose-dependent distortion of the thermal spectra profile in the 25–90°C temperature interval at PM doses of 0.2, 0.4, and 0.6 m*M* applied prior to the DSC analysis. The initial oxidative impact of the permanganate did not significantly change the general pattern of the melting curve. The dose increase did not cause the shift of the thermoabsorption peaks, but influenced their intensity. Comparing the changes in the thermogram shape, the sizes and positions of the peaks, and according to the analogy with the previous identifications[8,9,15], it could be suggested that the primary toxic effects of PM regarded membrane lipids, the onset of denaturation of the 30S ribosomal subunit, and soluble cytoplasmic proteins.

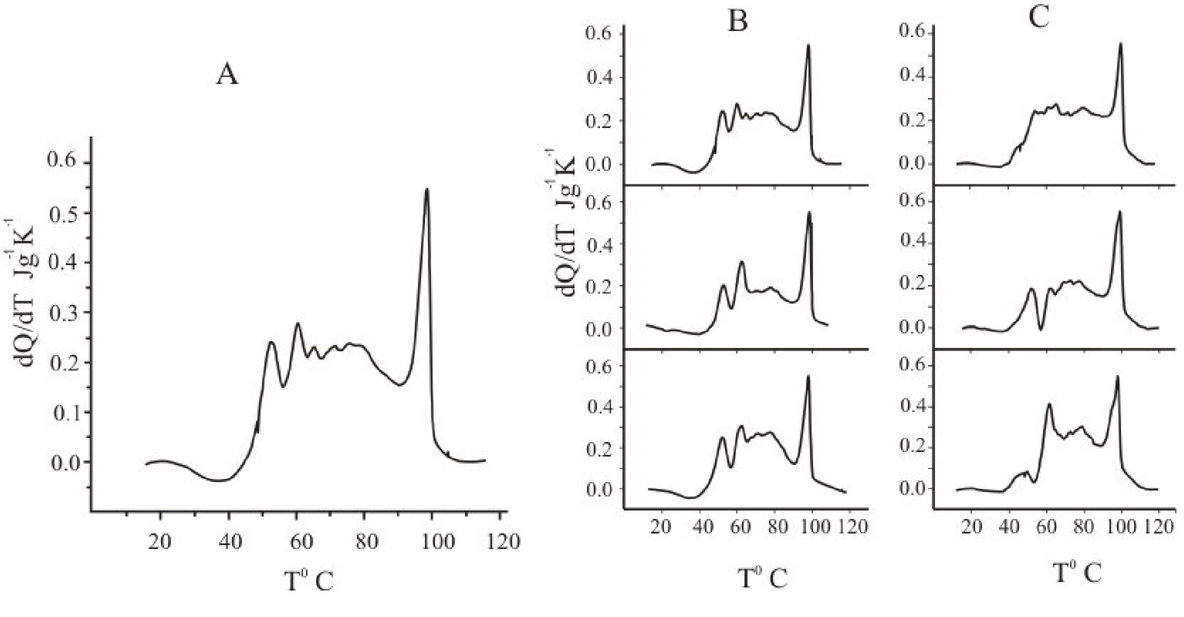

**FIGURE 1.** The time-dependent effect of different concentrations of permanganate on the thermal spectra of *A. oxydans*. (A) At 48 h of growth (stationary growth phase) as batch culture without permanganate. (B) At the permanganate application directly to the cell suspension at 48 h of growth prior to the calorimetry measurement (0.2, 0.4, 0.6 m*M*). (C) At the permanganate introduction into the culture media at 24 h of growth (stationary growth phase) with subsequent 24-h growth in the presence of the chemical and analyzed 48 h after cell inoculation (0.2, 0.4, 1.4 m*M*).

The time-dependent impact of permanganate (24-h cell growth in the presence of PM at different concentrations – 0.2, 0.4, and 1.4 m*M*) resulted in severe disorders of the thermal spectra profile over the whole temperature range 25–110°C (Fig. 1C). The complex events were possibly associated with the denaturation of ribosomes, components of the cell envelope, and also of DNA. Ribosome melting is a complex process occurring between 47 and 85°C. Peaks at 90–101°C result from the melting of a portion of the different region of cellular DNA combined with denaturation of a cell wall component[15].

Thermodynamic parameters of the processes are presented in Fig. 2 (A,B). The total heat of melting ($QJ/g_{dry\ mass}$) did not change significantly in a dose-dependent manner at the chemical application prior to the heat treatment at the calorimetry measurement (Fig. 2A). The time-dependent effect of permanganate treatment caused the increase of the total heat at the low concentration of 0.2 m*M* on about 10%. This rise of the parameter was followed by its gradual decline in accordance with the increase of the concentrations. The final toxic concentration of 1.4 m*M* cut off the total heat of melting on 30%.

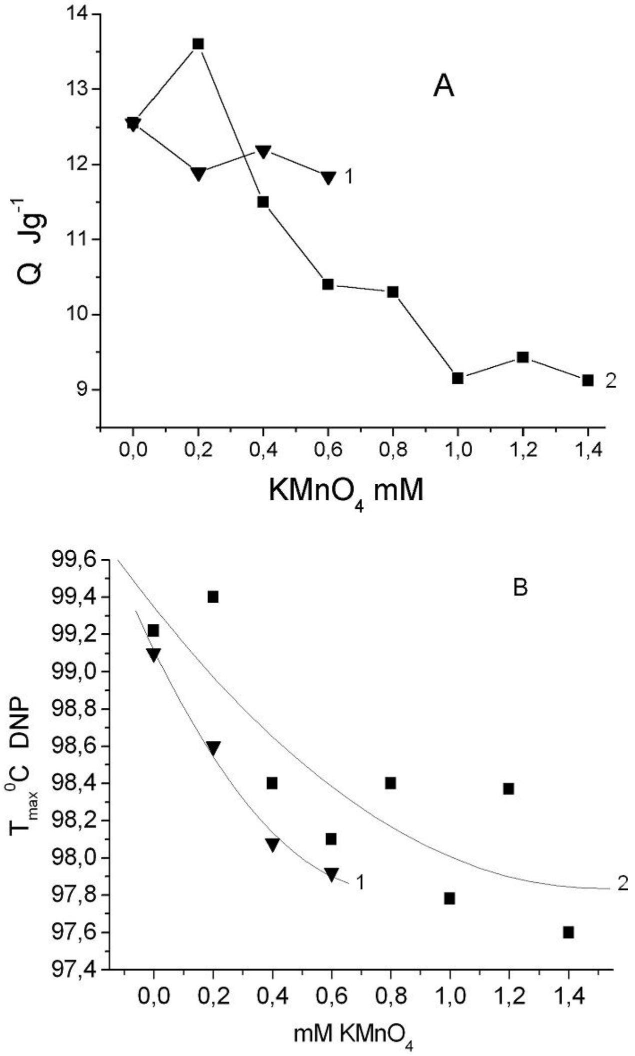

**FIGURE 2.** The time-dependent effect of different concentrations of permanganate on thermodynamic parameters. (A) The total heat of melting ($QJ/g_{dry\ mass}$). 1, Initial toxic effect of PM; 2, 24-h effect of PM. (B) The temperature of DNP melting ($T_{max}$ °C DNP). 1, Initial toxic effect of PM; 2, 24-h effect of PM.

The temperature of DNP melting ($T_{max}$ °C DNP) decreased in a dose- and time-dependent manner (Fig. 2B). The permanganate ion reacts strongly and rapidly with pyrimidine bases uracil and thymine, weakly and slowly with the pyrimidine base cytosine, and minimally with purine bases. $MnO_4^-$ forms an unstable cyclic permanganate ester with pyrimidine bases, resulting in their decomposition[16]. The toxic impact of permanganate on bacterial cells was revealed by the detection of a changed pattern of the melting curve, the appearance of some new thermoabsorption peaks in the temperature range 25–90°C, the lowered total heat (Fig. 2A), and the decreased temperature of DNP melting. These processes could reflect the destruction of intracellular components by the action of a broad-range oxidant, such as permanganate.

The influence of acid, ethanol, and salt on the cellular components and inactivation of *E. coli* were evaluated using DSC[8]. The thermal stability for ribosomal subunit denaturation and the total apparent enthalpy decreased with increasing ethanol, salt, and acid concentrations. The reduction of the ribosomal subunit denaturation peak (56–75°C) was the primary contributor to the decrease in the total apparent enthalpy. The effects of pH and ethanol were also more evident at lower temperatures of heat melting[17].

The low concentration of permanganate (0.2 m*M*) affected bacterial cells in an alternative way, raising the total heat on 10% and the temperature of DNP melting of 0.3 K, as had been previously detected at cell transition from the logarithmic to stationary growth stage.

The pattern of thermal spectra of *A. oxydans* at permanganate treatment, the total heat, and the temperature of DNP complex measurements provided the possibility for us to verify the permanganate impact on dependence of concentration values.

Cell viability was assessed by colony plate counting. The permanganate was administered into the culture media at concentrations 0.02, 0.2, 0.4, 0.6, 0.8, 1.0, and 1.2 m*M* at 24 h of cell growth (early stationary phase), and after the next 24 h of permanganate incubation, 100 µl of suspension were transferred onto agar plates. The effect of the studied oxidant on bacterial cell culture viability at 48 h was characterized by a biphasic dose-dependent response (Fig. 3). The low concentrations of $KMnO_4$ 0.02 and 0.2 m*M* stimulated cell proliferation, increasing the CFU parameter subsequently on 20 and 30%. The high concentrations of permanganate up to 0.4 m*M* caused the decline of cell viability. The PM concentration of 1.2 m*M* resulted in a 70% decrease of CFU/ml compared with control.

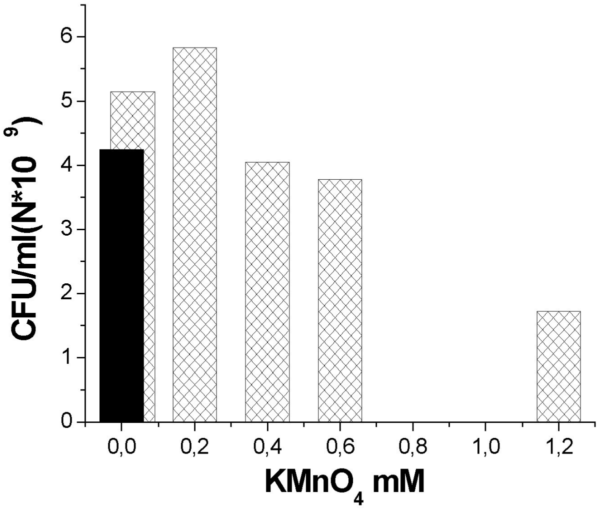

**FIGURE 3.** The effect of different concentrations of permanganate on cell viability, assessed by the colony forming ability. The permanganate was administered into the culture media at concentrations 0.02, 0.2, 0.4, 0.6, 0.8, 1.0, and 1.2 m*M* at 24 h of cell growth (early stationary phase) for 24 h.

The correlation between the enhanced growth of *A. oxydans* culture, the increased total heat, and the temperature of DNP complex melting at the impact of the low PM concentrations were analogical with the parameters at the logarithmic growth phase of the same bacterial cell culture[10].

The data presented in Fig. 4A demonstrate that the total melting heat of bacterial cells changes in accordance with the growth rate of the culture. The conditional graphical analysis of the whole bacterial cell melting spectra (Fig. 4B) regarding the different temperature ranges revealed the significant input to the value of the total cell heat of the melting heat of cell structures at the range 55–78°C (Fig. 4C). This region of thermograms had been connected with the state of the intracellular ribosomal system[8,15].

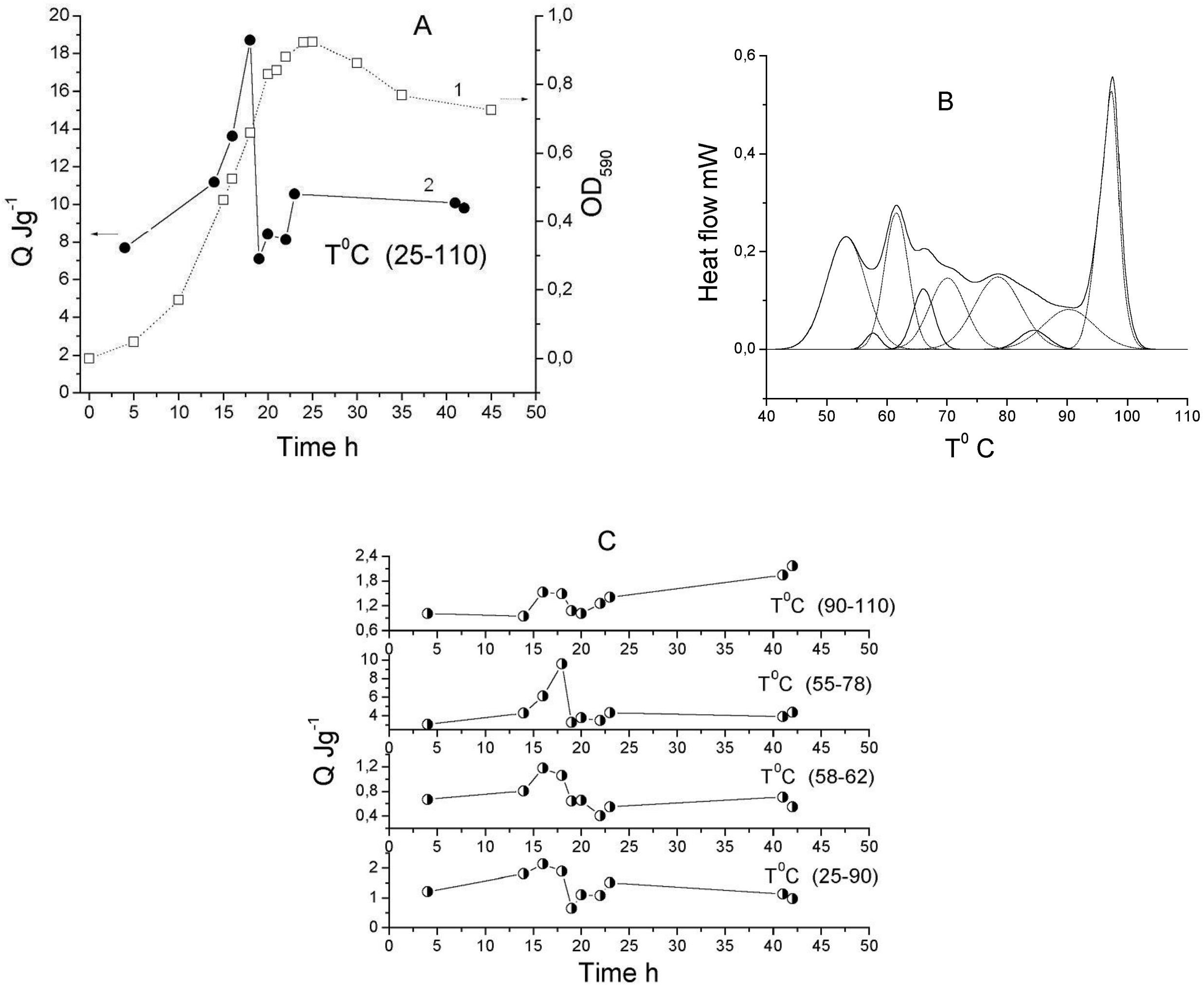

**FIGURE 4.** The correlation between the enhanced growth of *A. oxydans* culture and the increased total heat. (A) Bacterial cell culture transition from the logarithmic to stationary growth stage. 1, Bacterial cell culture growth curve; 2, total heat. (B) The conditional graphical analysis of the whole bacterial cell melting spectra at cell transition from the logarithmic to stationary growth stage. (C) The different temperature ranges of the whole bacterial cell melting spectra according the conditional graphical analysis.

A biphasic dose-response relationship, in which a chemical exerts opposite effects dependent on the dose and is characterized by low-dose stimulation and high-dose inhibition, has been frequently observed in different biological systems. It is broadly accepted as being independent of chemical/physical agent, biological model, and end-point measured[18,19,20].

Biological adaptive responses, resulting in the protection of cells against toxic agents, occur in response to low doses of all types of DNA-damaging agents[21,22]. Various physiological reactions, such as stimulation of the immune response, induction of detoxification, and repair enzymes, and up-regulation of tumor suppressor genes could result in paradoxical effects of low-dose exposure[23]. Potential mechanisms of such adaptive responses may be the activation of the antioxidant defense system, the activation of genes that can promote production of growth factors and cytokines, and DNA repair[24,25]. The mechanisms of such biphasic dose responses is not elucidated and the knowledge of these processes is fragmentary[26]. Mechanisms for responding to environmental changes are universally present in living beings. Bacterial cells are capable of persisting in diverse rapidly changing environmental conditions, sense the alterations, and respond by gene expression and protein activity[27], until the increased concentrations of toxic agents initiate the cell destructive processes.

The DSC technique applied to the study of the toxic impact of permanganate on a bacterial cell culture detected the lack of linearity in the dose-response effect. The results were confirmed by the traditional assay of colony forming ability. The changed pattern of thermal spectra of *A. oxydans* at permanganate treatment, the measurement of the total heat, and the temperature of the DNP complex demonstrate the possibility for us to verify the permanganate impact on dependence of concentrations value and to characterize the intracellular sequence events in response to toxic agent action, which initiates either a stress-adaptation-survival response or cell death, depending on the severity of the insult.

### ACKNOWLEDGMENTS

The authors would like to thank Dr. X.-Y. Holman (Lawrence Berkeley National Laboratory, Berkeley, CA) for her kind gift of the *A. oxydans* cell culture. We are grateful to the GNSF (Georgia) for Project 979/07 Grant research funding.
